# The Prognostic Value of HRAS mRNA Expression in Cutaneous Melanoma

**DOI:** 10.1155/2017/5356737

**Published:** 2017-11-19

**Authors:** Xiaohua Wan, Ruping Liu, Zhongwu Li

**Affiliations:** ^1^Department of Clinical Laboratory, Beijing Tongren Hospital, Capital Medical University, Beijing 100730, China; ^2^Beijing Institute of Graphic Communication, Beijing 102600, China; ^3^Key Laboratory of Carcinogenesis and Translational Research (Ministry of Education), Department of Pathology, Peking University Cancer Hospital & Institute, Beijing 100142, China

## Abstract

This study aimed to investigate the prognostic value of HRAS mRNA expression in cutaneous melanoma. Cutaneous melanoma is an aggressive cancer with an increasing incidence. Few studies have focused on the transcriptional level of RAS isoforms (KRAS, NRAS, and HRAS) in cutaneous melanoma. To gain further insight into RAS isoforms at transcriptional level, we obtained the cutaneous melanoma data from cBioPortal and investigated the RAS mRNA expression levels in different stages of melanoma and evaluated their correlation with clinical characteristics and patients' survival. Furthermore, we retrieved and analyzed the coexpression data and performed pathway enrichment analysis. Totally, 452 cutaneous melanoma cases were included in this study. We found that lower HRAS expression level was associated with longer patient survival. 206 genes that negatively correlated with HRAS expression were positively correlated with KRAS and NRAS expression. In contrast, no gene that positively correlated with HRAS expression was positively correlated with KRAS and NRAS expression. In conclusion, our data showed that transcriptional regulation was different for the three RAS isoforms in cutaneous melanoma. This study highlighted the prognostic value of HRAS mRNA expression and revealed that HRAS greatly differs from KRAS and NRAS at the transcriptional level.

## 1. Introduction

RAS proteins are small GTPases that signal downstream of cell surface receptors to regulate cell proliferation, differentiation, migration, and apoptosis [1–3]. In human, there are 3 ubiquitously expressed RAS genes, KRAS, NRAS, and HRAS, which encode four highly homologous RAS proteins (splicing variants KRAS4A/4B, NRAS, and HRAS). These RAS genes are the most frequently mutated genes in human cancers [4]. Mutations in the RAS genes are associated with around 30% of all human tumors [5]. In a specific type of cancer, usually only a single RAS isoform is mutated. For example, mutations in KRAS are common in lung, colon, and pancreatic cancers, those in NRAS predominate in melanoma, and HRAS mutations are commonly seen in bladder, head and neck, and skin cancers [6–9].

Cutaneous melanoma is an aggressive cancer with an increasing incidence [10]. Alterations in multiple signaling pathways that regulate cell proliferation and survival contribute to the tumorigenesis and progression of this disease [11]. Cutaneous melanoma frequently harbors activating mutations in NRAS (around 20%) or the RAS-regulated kinase BRAF (around 37%), suggesting that the RAS-RAF-MAPK pathway may be critical in the pathogenesis of cutaneous melanoma [12–14]. Although NRAS shares high sequence homology with KRAS and HRAS and all these three RAS isoforms can be signaled through the RAS-RAF-MAPK pathway, HRAS and KRAS mutations are rare in this disease [15, 16]. Different functions of these three RAS isoforms have been reported in many literatures [1, 17–19]. However, differential regulating of these RAS isoforms at transcriptional level was not well studied.

Currently, there has been remarkable progress in understanding melanoma pathogenesis, and numerous studies have now shown the correlation of BRAF and NRAS mutation status with clinical outcome and immune and targeted therapy strategies in melanoma [13, 20]. However, few studies have focused on the characteristics of RAS isoforms mRNA expression in cutaneous melanoma.

In this study, we sought to examine the differential regulation of RAS isoforms at mRNA level in cutaneous melanoma. We analyzed the correlation between RAS mRNA expression and different clinical characteristics and explored the genetic mutation data. By analyzing genes coexpressed with different RAS isoforms, we found significant difference between genes coexpressed with HRAS and those with KRAS/NRAS. We also analyzed the correlation of RAS mRNA expression with BRAF. Furthermore, we performed pathway enrichment analysis, which provided more clues about the differential regulation of RAS isoforms.

## 2. Materials and Methods

### 2.1. Data Sources and Bioinformatics

We obtained the cutaneous melanoma data from cBioPortal (http://www.cbioportal.org). The data of skin cutaneous melanoma (TCGA, Provisional) included mRNA expression data (RNA Seq V2 RSEM) of the three RAS isoforms (KRAS, NRAS, and HRAS), matching clinical information, genetic mutation data, and genes coexpressed with the three RAS isoforms. The cases selection included three criteria: (i) each patient ID corresponded to a unique sample ID; (ii) the case included available RAS isoforms (KRAS, NRAS, and HRAS) mRNA expression data; and (iii) the case included available age and gender information.

The following variables were obtained from cBioPortal (http://www.cbioportal.org): age, gender, AJCC stage, tumor thickness, ulceration, overall survival status, genetic mutation data, and genes coexpressed with the three RAS isoforms. To ensure accuracy, dual data extraction was conducted. Data were subsequently verified between the 2 reviewers, and discrepancies were settled through consensus discussion. To minimize subjective judgment and selection bias, investigators were blinded to outcomes.

### 2.2. Clinical Characteristics and Survival Analysis

We compared mRNA expression levels of the three RAS isoforms in different clinical subgroups. Clinical factors considered included age, gender, AJCC stage, tumor thickness (mm), and ulceration. Cases were divided into “low” and “high” groups based on the median RAS expression level.

For survival analysis, based on the median levels of RAS mRNA expression, cases were grouped into “low” and “high.” Because BRAF is one of the most important genes in melanoma, we also investigated the correlation of BRAF mRNA expression and overall survival. Cases were also divided into “low” and “high” groups based on the median BRAF expression level.

Because LCK was an important prognostic factor in cutaneous melanoma [21], we also analyzed the correlation between LCK and HRAS at transcriptional level.

### 2.3. mRNA Expression of RAS Isoforms according to Genetic Mutation Status

We compared RAS mRNA expression levels according to RAS mutation status and BRAF mutation status, as BRAF is one of the most frequently mutated genes in cutaneous melanoma. Additionally, we analyzed the correlation of RAS mRNA expression and BRAF mRNA expression.

### 2.4. Coexpression and Pathway Enrichment Analysis

We retrieved and analyzed the coexpression data using the coexpression tool in cBioPortal. Genes with Pearson's correlation coefficients (CC) ≥ 0.3 were considered positively correlated with RAS expression. Genes with Pearson's correlation coefficients (CC) ≤ −0.3 were considered negatively correlated with RAS expression. For the genes positively and negatively correlated with KRAS, NRAS, and HRAS expression, we analyzed the number of overlapping genes and enriched pathways.

The pathway enrichment analysis was carried out using the default parameters of Kyoto Encyclopedia of Genes and Genomes (KEGG) pathway by the Database for Annotation Visualization and Integrated Discovery (DAVID) (https://david.ncifcrf.gov/). *p* < 0.05 was used as the cut-off.

### 2.5. Statistical Analysis

The correlations between RAS mRNA expression and clinical characteristics including age, gender, AJCC stage, tumor thickness, and ulceration were analyzed by the Chi-square test. Overall survival was assessed by Kaplan-Meier analysis, and univariate and multivariate Cox regression analyses were used to calculate hazard ratios (HRs) and their 95% confidence intervals (95% CI). The RAS mRNA expression difference between wild-type and mutant groups was evaluated by Mann–Whitney test. In addition, Pearson's correlation coefficients analysis was used for the correlation of LCK and HRAS, RAS isoforms, and BRAF at transcriptional level, respectively. All tests were 2-sided, and *p* < 0.05 was considered statistically significant. All analysis was conducted using SPSS 17.0 (IBM Corporation, Armonk, NY, USA).

## 3. Results

### 3.1. Clinicopathological and Demographic Characteristics of Patients

A total 452 cutaneous melanoma patients were included in this study, with 280 males and 172 females. All cases had available age, gender, and mRNA expression data for the three RAS isoforms by RNA Seq V2 RSEM, and each patient ID corresponded to a unique sample ID. The data were obtained on August 22, 2016.

The median age was 58 years (range, 15~90 years), and the medium overall survival follow-up time was 36.93 months (range, 0.20~369.65 months). The baseline clinicopathological and demographic characteristics of patients were summarized in Table 1. A total of 216 patients (47.8%) died during the period of follow-up.

### 3.2. The Relationship between mRNA Expression of RAS Isoforms and Clinical Characteristics

The correlation between RAS mRNA expression and clinical characteristics was summarized in Table 2. There was significant correlation between HRAS mRNA expression and tumor thickness; higher HRAS mRNA level was correlated with higher tumor thickness (*p* = 0.002). There was no correlation between HRAS mRNA expression and age, gender, AJCC stage, and ulceration, respectively. KRAS (*p* = 0.048) and NRAS (*p* = 0.048) mRNA expressions were positively correlated with age, respectively. No other significant correlations between KRAS or NRAS mRNA expression and gender, AJCC stage, tumor thickness, or ulceration were found (Table 2).

### 3.3. Prognostic Value of HRAS mRNA Expression in Cutaneous Melanoma

**Figure 1:**
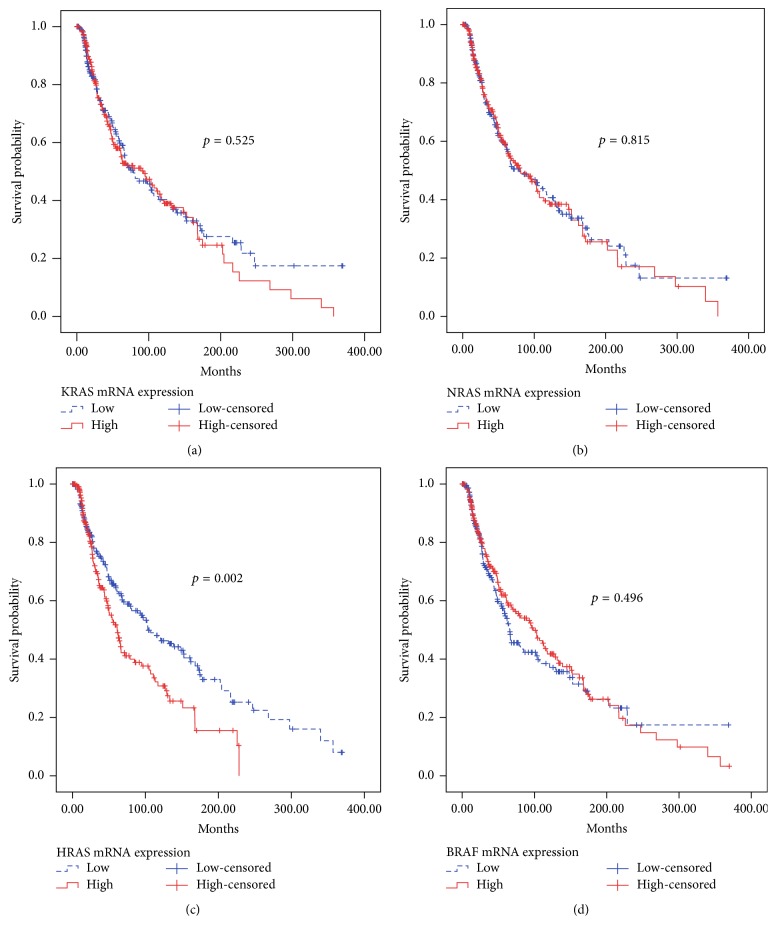
*The correlation between KRAS, NRAS, HRAS, and BRAF mRNA expression and patients' overall survival in cutaneous melanoma, respectively*. (a) No significant correlation was observed in KRAS mRNA expression and patients' overall survival (*p* = 0.525). (b) No significant correlation was observed in NRAS mRNA expression and patients' overall survival (*p* = 0.815). (c) Patients with low HRAS (*p* = 0.002) mRNA expression had longer overall survival than those with high expression. (d) No significant correlation was observed in BRAF mRNA expression and patients' overall survival (*p* = 0.496).

Interestingly, lower HRAS expression level was associated with longer overall survival (Figure 1(c)). Cases were classified into “low” and “high” groups using median HRAS mRNA expression as cut-off. For total 452 cases, time to death was plotted in a Kaplan-Meier curve for those cases exhibiting HRAS mRNA expression above the median (*n* = 226) or below the median (*n* = 226) level of expression. As shown in Figure 1(c), the survival curves were significantly different (*p* = 0.002); those cases had lower mRNA expression of HRAS, surviving longer (Figure 1(c)). Furthermore, our results showed that there was a negative correlation between LCK and HRAS at transcriptional level (CC = −0.122; *p* = 0.01).

Similar overall survival analyses were carried out for KRAS, NRAS, and BRAF mRNA expression. There were no significant differences in the relationships of KRAS (*p* = 0.525, Figure 1(a)), NRAS (*p* = 0.815, Figure 1(b)), and BRAF (*p* = 0.496, Figure 1(d)) mRNA expression and overall survival when total cases (*n* = 452) were considered, respectively.

In addition, we used a Cox proportional hazard regression model to estimate the crude HRs of each clinicopathological characteristic (Table 3). Univariate Cox regression survival analysis showed that high HRAS mRNA expression was a risk factor for worse patient survival (HR: 1.532, 95% CI: 1.163~2.017, *p* = 0.002 for overall survival). And univariate analysis revealed that age (*p* < 0.001), AJCC stage (*p* = 0.002), tumor thickness (*p* < 0.001), and ulceration (*p* < 0.001) were all significantly associated with overall survival (Table 3). However, KRAS (*p* = 0.525), NRAS (*p* = 0.815), and BRAF (*p* = 0.496) mRNA expressions did not correlate with patient survival (Table 3). Furthermore, multivariate Cox regression analyses on overall survival showed that HRAS mRNA expression (HR: 1.555, 95% CI: 1.066~2.269, *p* = 0.022), AJCC stage (HR: 1.921, 95% CI: 1.336~2.762, *p* < 0.001), and ulceration (HR: 1.787, 95% CI: 1.229~2.600, *p* = 0.002) were independent prognostic factors.

### 3.4. mRNA Expression of RAS Isoforms according to Genetic Mutation Status

In 452 patients, 182 (40.3%) melanomas were BRAF-mutant, 95 (21.0%) were NRAS-mutant, and 180 (39.8%) were wild-type (no BRAF mutation and no NRAS mutation); only 5 (1.1%) were both BRAF-mutant and NRAS-mutant. There are very few cases of KRAS mutation (7 cases, 1.5%) or HRAS mutation (5 cases, 1.1%) (Table 4).

Compared to wild-type RAS isoforms, mutational RAS isoforms mRNA expressions were significantly higher, respectively (*p* = 0.005 for KRAS, *p* < 0.001 for NRAS, and *p* = 0.030 for HRAS) (Table 4). For the NRAS-mutant group, HRAS mRNA expression was significantly lower than NRAS wild-type group (*p* < 0.001) (Table 4). For the group with BRAF mutation, NRAS mRNA expression was significantly lower than BRAF wild-type group (*p* = 0.015) (Table 4).

**Figure 2:**
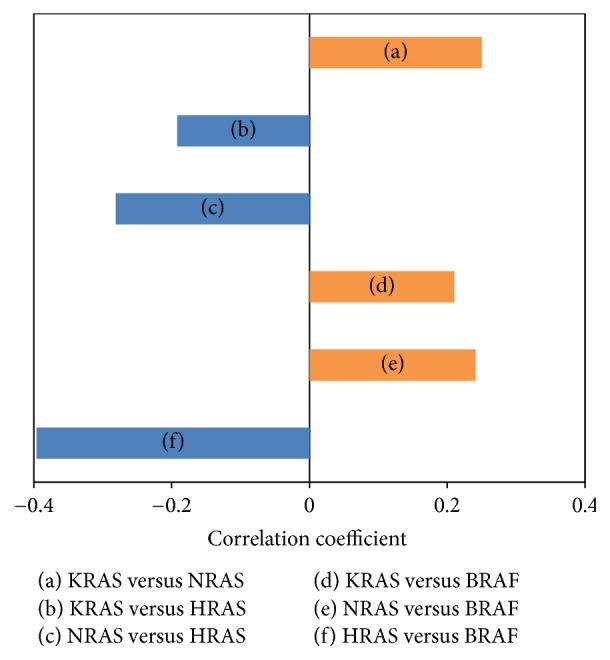
*Correlation between mRNA expression of RAS isoforms and BRAF*. There was a positive correlation between KRAS and NRAS (CC = 0.250; *p* < 0.001), between KRAS and BRAF (CC = 0.210; *p* < 0.001), and between NRAS and BRAF (CC = 0.241; *p* < 0.001) mRNA expressions, respectively. It was noted that HRAS mRNA expression was negatively correlated with KRAS (CC = −0.192; *p* < 0.001), NRAS (CC = −0.281; *p* < 0.001), and BRAF (CC = −0.396; *p* < 0.001) mRNA expressions, respectively.

Furthermore, we have analyzed the correlation of RAS mRNA expression and BRAF mRNA expression. The results showed that there was a positive correlation between KRAS and NRAS (CC = 0.250; *p* < 0.001), between KRAS and BRAF (CC = 0.210; *p* < 0.001), and between NRAS and BRAF (CC = 0.241; *p* < 0.001), respectively. Notably, HRAS mRNA expression was negatively correlated with KRAS (CC = −0.192; *p* < 0.001), NRAS (CC = −0.281; *p* < 0.001), and BRAF (CC = −0.396; *p* < 0.001) mRNA expressions, respectively (Figure 2).

### 3.5. Coexpression and Pathway Enrichment Analysis

**Figure 3:**
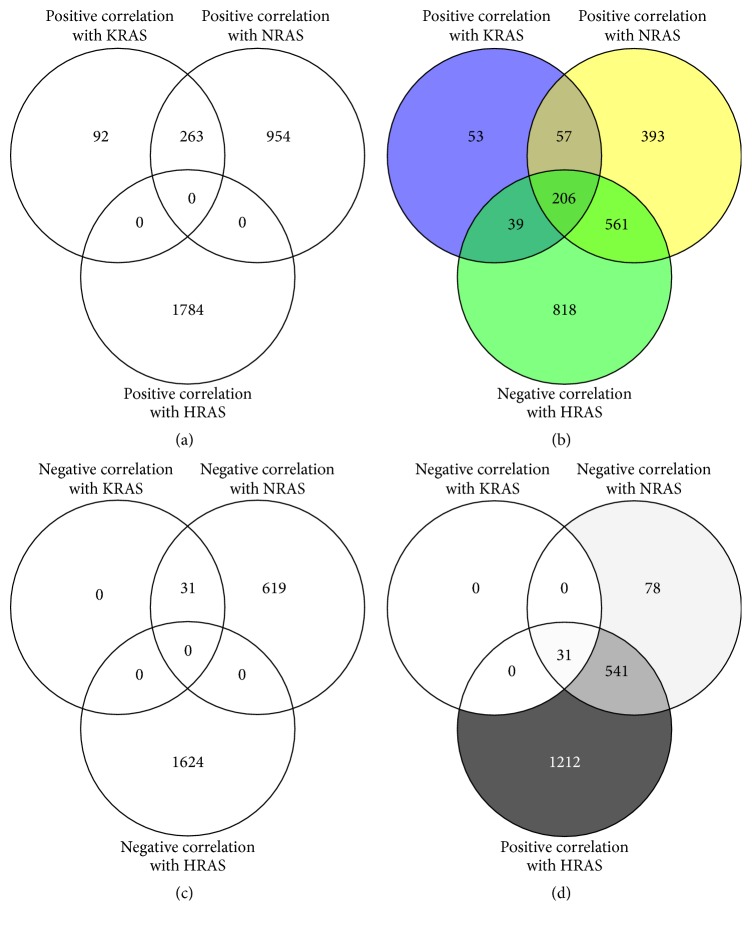
*The number of overlapping genes correlated with RAS mRNA expression*. ((a) and (b)) The number of overlapping genes positively correlated with KRAS and NRAS expression and positively or negatively correlated with HRAS expression. ((c) and (d)) The number of overlapping genes negatively correlated with KRAS and NRAS expression and positively or negatively correlated with HRAS expression.

To get a better picture about transcriptional regulation of RAS isoforms, we analyzed genes that were coexpressed with RAS isoforms. Genes with Pearson's correlation coefficient (CC) ≥ 0.3 or ≤−0.3 were evaluated. 355 genes were positively correlated and 31 were negatively correlated with KRAS expression. 1217 genes were positively correlated and 650 were negatively correlated with NRAS expression. There were 1784 genes positively correlated and 1624 genes negatively correlated with HRAS expression (Figure 3).

**Figure 4:**
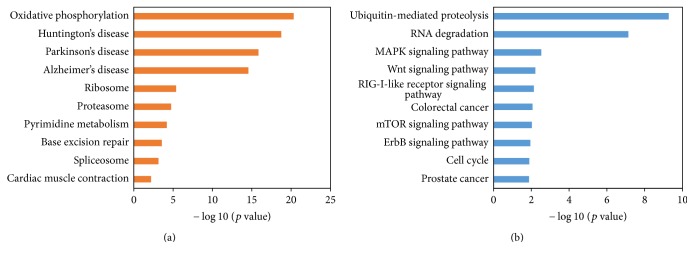
The top 10 enriched pathways by KEGG pathway analyses for genes positively and negatively correlated with HRAS expression. (a) The top 10 enriched pathways for genes positively correlated with HRAS expression. (b) The top 10 enriched pathways for genes negatively correlated with HRAS expression.

In addition, we explored the top 10 enriched pathways by KEGG pathway analyses for genes positively (Figure 4(a)) and negatively (Figure 4(b)) correlated with HRAS expression, respectively. The enriched pathways of the genes positively correlated with HRAS included oxidative phosphorylation (hsa00190, *p* = 4.73*E* − 21), Huntington's disease (hsa05016, *p* = 1.78*E* − 19), Parkinson's disease (hsa05012, *p* = 1.42*E* − 16), Alzheimer's disease (hsa05010, *p* = 2.70*E* − 15), and ribosome (hsa03010, *p* = 4.11*E* − 6) (Figure 4(a)). And the enriched pathways of the genes negatively correlated with HRAS included ubiquitin-mediated proteolysis (hsa04120, *p* = 5.32*E* − 10), RNA degradation (hsa03018, *p* = 7.29*E* − 8), MAPK signaling pathway (hsa04010, *p* = 0.003), Wnt signaling pathway (hsa04310, *p* = 0.006), and RIG-I-like receptor signaling pathway (hsa04622, *p* = 0.007) (Figure 4(b)).

Interestingly, 206 genes that negatively correlated with HRAS expression were positively correlated with KRAS and NRAS expression. In contrast, no gene that positively correlated with HRAS expression was positively correlated with KRAS and NRAS expression (Figures 3(a) and 3(b)).

To explore possible signal pathways, we studied the enriched pathways of the 206 genes that negatively correlated with HRAS but positively correlated with KRAS and NRAS expression (Table 5). We found that these genes were enriched in the following pathways (*p* < 0.05): chronic myeloid leukemia (hsa05220, *p* = 0.004), prostate cancer (hsa05215, *p* = 0.007), regulation of actin cytoskeleton (hsa04810, *p* = 0.011), acute myeloid leukemia (hsa05221, *p* = 0.014), cell cycle (hsa04110, *p* = 0.024), spliceosome (hsa03040, *p* = 0.024), pancreatic cancer (hsa05212, *p* = 0.025), MAPK signaling pathway (hsa04010, *p* = 0.029), and TGF-beta signaling pathway (hsa04350, *p* = 0.041) (Table 5).

Similar analyses were carried out for genes positively correlated with HRAS expression and negatively correlated with KRAS/NRAS mRNA expression. There were 31 genes in this group, but no gene that negatively correlated with KRAS, NRAS, and HRAS was found (Figures 3(c) and 3(d)). For the 31 overlapping genes, no pathway was significantly associated with these genes.

## 4. Discussion

This study highlighted a novel role for the prognostic value of HRAS mRNA expression in cutaneous melanoma. To our knowledge, few studies examined the association of RAS mRNA expression with overall survival. Our data indicated that HRAS mRNA expression, but not KRAS or NRAS, was correlated with prognosis in cutaneous melanoma. Patients with higher HRAS mRNA expression levels in the tumors had poorer overall survival.

It has been reported that HRAS mRNA level has prognostic meaning in triple-negative breast cancers [22]. In our study, we found that HRAS mRNA level was correlated with the poor prognosis of the cutaneous melanoma. We also detected that increased HRAS mRNA level was correlated with high thickness. More mechanistic studies are needed to further elucidate the relationship between HRAS mRNA and the prognosis of cutaneous melanoma [23]. Furthermore, Because LCK was the most important prognostic factor in cutaneous melanoma [21], the negative correlation between HRAS and LCK at transcriptional level indicates that HRAS might be a critical molecule in cutaneous melanoma.

Whether RAS mutation is a prognostic factor in melanoma was controversial. Some studies found no prognostic impact of mutation status [24] and other studies demonstrated that BRAF-mutant or NRAS-mutant melanoma patients had poorer overall survival [25–27].

Another interesting observation is that HRAS mRNA expression was negatively correlated with that of KRAS, NRAS, and BRAF, respectively. In contract, KRAS, NRAS, and BRAF mRNA expressions were positively correlated with each other. We also found that there were 206 genes that negatively correlated with HRAS expression significantly overlapped with genes positively correlated with KRAS/NRAS in cutaneous melanoma. Coexpression analysis showed that there was no gene coexpressed with all three isoforms of RAS. This observation suggests that regulation of HRAS in melanoma is different from that of KRAS and NRAS. Although those three RAS isoforms share similar downstream pathways, their transcriptional regulation may be different.

Despite a high degree of sequence homology among the RAS isoforms, different functions and subcellular localization of them have been reported [3]. Different RAS isoforms distinctly contribute to embryonic development, cancer development, cellular homeostasis, and differential coupling to canonical effector pathways [3, 28]. However, the mechanisms underlying the biological differences between the RAS isoforms remain unclear.

Our study suggests that, at transcriptional level, the three RAS genes are differently regulated. RAS isoforms have some distinct transcription factors. Transcription factor binding site analysis in the promoter region of HRAS suggests that p53, STAT3, c-Myc, NF-1, NF-1/L, NF-*κ*B, NF-Y, c-Myb, and Max can regulate HRAS transcription. For KRAS, the transcription factors include p53, AP-1, c-Jun, Elk-1, NF-*κ*B, STAT1, and PPAR-*γ*1. For NRAS, the transcription factors are p53, C/EBP-*α*, c-Fos, AP-1, c-Jun, STAT5A, MyoD, and NF-1 (data obtained from GeneCards: the Human Gene Database; http://www.genecards.org/). There are 47 NF-1 binding sites in the HRAS gene promoter but much less for KRAS and NRAS. In KRAS gene promoter, there are 16 binding sites of transcription factor STAT1, which are the most abundant. In NRAS gene promoter, there are 11 binding sites of AP-1, which is the most abundant transcription factor (data obtained from GeneCards: the Human Gene Database; http://www.genecards.org/). In the pathogenesis of cutaneous melanoma, the differential regulation of the above-mentioned transcription factors may be the primary reason to regulate RAS isoforms mRNA expression.

On average, 16% of human cancers harbor activating mutations of RAS at amino acid 12, 13, or 61 [29]. With regard to NRAS, the most common oncogenic change (>80% of all NRAS mutations) is a point mutation leading to the substitution of leucine by glutamine at amino acid 61, with mutations at amino acids 12 and 13 occurring less frequently [8, 14]. Amino acid 61 mutations also account for the majority of HRAS mutations in melanoma, whereas most KRAS mutations are at amino acid 12 [14]. Although it is not clear why NRAS mutations are more frequent in melanoma compared to HRAS or KRAS mutations, there is evidence that NRAS is overexpressed in melanocytes relative to other RAS isoforms. It is also possible that NRAS may activate different signaling pathways from KRAS and HRAS, an idea supported by the observation that NRAS has greater transforming activity than KRAS in experimental models of melanoma [14, 15, 30].

RAS activating mutations have been viewed as functionally dominant because they are constitutively active. However, the status of the wide-type RAS allele may also play a role in tumors carrying mutant RAS genes [9, 30]. Several recent studies have shown that oncogenic and wild-type RAS play independent and nonredundant roles within the cell. Oncogenic RAS regulates basal effector signaling, whereas wild-type RAS mediates signaling downstream of activated receptor tyrosine kinases (RTKs) [30, 31]. Further studies are needed to determine the association between wild-type RAS and mutants.

## 5. Conclusions

In conclusion, our data showed that transcriptional regulation was different for the three RAS isoforms in cutaneous melanoma. This study highlighted the prognostic value of HRAS mRNA expression and revealed that HRAS greatly differs from KRAS and NRAS at the transcriptional level. Further understanding of the upstream signaling pathways regulating RAS gene expression will shed light on pathogenesis of cutaneous melanoma.

## Figures and Tables

**Table 1:** Clinicopathological and demographic characteristics of patients with cutaneous melanoma (TCGA, Provisional).

Characteristics	Cases number	Percentage (%)
Age		
≤58	227	50.2
>58	225	49.8
Gender		
Male	280	61.9
Female	172	38.1
AJCC stage		
Stage I	75	16.6
Stage II	139	30.8
Stage III	167	36.9
Stage IV	21	4.6
Unknown	50	11.1
Tumor thickness (mm)		
≤1.00	57	12.6
1.01~2.00	77	17.0
2.01~4.00	68	15.0
>4.00	146	32.3
Unknown	104	23.0
Ulceration		
Absent	142	31.4
Present	163	36.1
Unknown	147	32.5
Overall survival		
Living	236	52.2
Deceased	216	47.8

**Table 2:** The relationship between RAS mRNA expression and clinical characteristics of patients with cutaneous melanoma (TCGA, Provisional).

Characteristics	Cases number	KRAS mRNA expression			NRAS mRNA expression			HRAS mRNA expression		
		Low (%)	High (%)	*p* value	Low (%)	High (%)	*p* value	Low (%)	High (%)	*p* value
Age										
≤58	227	124 (54.6)	103 (45.4)	0.048	124 (54.6)	103 (45.4)	0.048	121 (53.3)	106 (46.7)	0.158
>58	225	102 (45.3)	123 (54.7)		102 (45.3)	123 (54.7)		105 (46.7)	120 (53.3)	
Gender										
Male	280	145 (51.8)	135 (48.2)	0.333	134 (47.9)	146 (52.1)	0.245	134 (47.9)	146 (52.1)	0.245
Female	172	81 (47.1)	91 (52.9)		92 (53.5)	80 (46.5)		92 (53.5)	80 (46.5)	
AJCC stage										
Stages I + II	214	108 (50.5)	106 (49.5)	0.759	104(48.6)	110 (51.4)	0.862	100 (46.7)	114 (53.3)	0.366
Stages III + IV	188	92 (48.9)	96 (51.1)		93 (49.5)	95 (50.5)		96 (51.1)	92 (48.9)	
Tumor thickness (mm)										
≤2.00	134	60 (44.8)	74 (55.2)	0.061	57 (42.5)	77 (57.5)	0.078	80 (59.7)	54 (40.3)	0.002
>2.00	214	119 (55.6)	95 (44.4)		113 (52.8)	101 (47.2)		91 (42.5)	123 (57.5)	
Ulceration										
Absent	142	72 (50.7)	70 (49.3)	0.801	62 (43.7)	80 (56.3)	0.139	73 (51.4)	69 (48.6)	0.248
Present	163	85 (52.1)	78 (47.9)		85 (52.1)	78 (47.9)		73 (44.8)	90 (55.2)	

**Table 3:** Univariate and multivariate Cox regression analyses on overall survival of patients with cutaneous melanoma (TCGA, Provisional).

Variable	Univariate		Multivariate	
	HR (95% CI)	*p* value	HR (95% CI)	*p* value
Age (>58 vs. ≤58)	1.674 (1.266~2.215)	<0.001	1.232 (0.854~1.779)	0.265
Gender (female versus male)	1.138 (0.856~1.513)	0.347	1.066 (0.742~1.531)	0.729
AJCC stage (III/IV versus I/II)	1.589 (1.182~2.137)	0.002	1.921 (1.336~2.762)	<0.001
Tumor thickness (>2 mm versus ≤2 mm)	2.237 (1.626~3.076)	<0.001	1.309 (0.881~1.947)	0.183
Ulceration (present versus absent)	2.140 (1.529~2.996)	<0.001	1.787 (1.229~2.600)	0.002
Gene mRNA expression				
KRAS (high versus low)	1.091 (0.834~1.428)	0.525	0.915 (0.634~1.319)	0.633
NRAS (high versus low)	1.033 (0.789~1.350)	0.815	0.981 (0.678~1.419)	0.981
HRAS (high versus low)	1.532 (1.163~2.017)	0.002	1.555 (1.066~2.269)	0.022
BRAF (high versus low)	0.910 (0.695~1.193)	0.496	0.794 (0.552~1.143)	0.215

**Table 4:** mRNA expression levels of RAS isoforms according to genetic mutation status of patients with cutaneous melanoma (TCGA, Provisional) (median (95% CI)).

Gene (cases)	KRAS		NRAS		HRAS	
	mRNA expression	*p* Value	mRNA expression	*p* Value	mRNA expression	*p* Value
*KRAS status*						
Wild-type (445)	667.79 (137.53~1487.34)	0.005	1597.28 (384.98~3956.68)	0.790	659.44 (272.77~2489.36)	0.725
Mutant (7)	1084.28 (459.27~2184.22)		1579.45 (501.62~3304.27)		668.81 (365.36~1149.48)	
*NRAS status*						
Wild-type (357)	667.47 (135.30~1532.54)	0.356	1440.21 (364.62~3183.51)	<0.001	680.69 (304.00~2568.04)	<0.001
Mutant (95)	683.78 (240.27~1524.89)		2273.43 (862.43~7465.98)		572.90 (144.64~1820.63)	
*HRAS status*						
Wild-type (447)	669.60 (143.99~1519.00)	0.792	1597.28 (413.96~3950.88)	0.339	658.60 (272.92~2258.13)	0.030
Mutant (5)	734.55 (97.39~756.41)		1120.72 (366.42~2219.04)		2147.36 (276.85~3769.50)	
*BRAF status*						
Wild-type (270)	675.86 (139.36~1644.12)	0.753	1665.42 (376.97~4491.40)	0.015	694.15 (258.75~2615.07)	0.100
Mutant (182)	670.57 (135.98~1482.67)		1517.62 (392.64~3439.89)		628.72 (277.64~2247.95)	

**Table 5:** The enriched pathways of the 206 genes that negatively correlated with HRAS but positively correlated with KRAS and NRAS expression.

Term	KEGG pathway	*p* value
hsa05220	Chronic myeloid leukemia	0.004
hsa05215	Prostate cancer	0.008
hsa04810	Regulation of actin cytoskeleton	0.011
hsa05221	Acute myeloid leukemia	0.014
hsa04110	Cell cycle	0.024
hsa03040	Spliceosome	0.024
hsa05212	Pancreatic cancer	0.025
hsa04010	MAPK signaling pathway	0.029
hsa04350	TGF-beta signaling pathway	0.041
